# The importance of APC

**DOI:** 10.1186/1740-2557-2-3

**Published:** 2005-04-26

**Authors:** Vitaly Ablamunits

**Affiliations:** 1 Department of Medicine, St. Luke's-Roosevelt Hospital Center, New York, NY, USA; 2 Institute for Human Nutrition, Columbia University, New York, NY, USA

## Abstract

Readers in immunology are familiar with the importance of antigen presenting cells in mounting immune responses. For the purpose of this particular editorial article, however, the abbreviation APC will stand for article processing charges. The publisher will introduce APCs for this *Journal *in May, 2005. Here we explain why article-processing charges are important to maintain our Open Access journal.

## 

*Journal of Autoimmune Diseases *is published by BioMed Central, an independent publisher committed to ensuring peer-reviewed biomedical research is Open Access – universally and freely available online to everyone, with authors retaining copyright, and the full text being archived in numerous internationally recognised free repositories [1]. *Journal of Autoimmune Diseases *however, has taken this further, by making *all *its content Open Access. To fund this, authors of articles accepted for publication in the *Journal *will be asked to pay an article-processing charge (APC). In 2005, that charge will be 330 GBP.

Traditionally, readers pay to access articles, either through subscriptions or by paying a fee each time they download an article. Escalating journal subscription prices have resulted in libraries subscribing to fewer journals [2], and the range of articles available to readers is therefore limited. Although traditional journals publish authors' work for free (unless there are page or colour charges), having to pay to access articles limits how many can read, use and cite them.

*Journal of Autoimmune Diseases' *Open Access policy changes the way in which articles are published. First, all articles become freely and universally accessible online, and so an author's work can be read by anyone at no cost. Second, the authors hold copyright for their work and grant anyone the right to reproduce and disseminate the article, provided that it is correctly cited and no errors are introduced [1]. Third, a copy of the full text of each article is permanently archived in a number of online repositories separate from the journal. Articles published in *Journal of Autoimmune Diseases *are archived in PubMed Central [3], the US National Library of Medicine's full-text repository of life science literature, and also in repositories at the University of Potsdam [4] in Germany, at INIST [5] in France and in e-Depot [6], the National Library of the Netherlands' digital archive of all electronic publications.

## Who benefits from the Open Access?

Open Access has four broad benefits for science and the general public. First, authors are assured that their work is disseminated to the widest possible audience, given that there are no barriers to access their work. It has been shown that free online articles are more highly cited because of their easier availability [7]. Second, the information available to researchers will not be limited by what their library can afford, and the widespread availability of articles will enhance literature searching [8]. Third, the results of publicly funded research will be accessible to all taxpayers and not just those with access to a library with a subscription. Note that this public accessibility may become a legal requirement in the USA if the proposed Public Access to Science Act is made law [9]. Fourth, a country's economy will not influence its scientists' ability to access articles because resource-poor countries (and institutions) will be able to read the same material as wealthier ones (although creating access to the internet is another matter [10]). APC will allow continued Open Access to all of the articles published in *Journal of Autoimmune Diseases*.

## What does APC cover?

The APC pays for the article to be freely and universally accessible in various formats online, and for the processes required for inclusion in PubMed and archiving in PubMed Central, e-Depot, Potsdam and INIST. Although some authors may consider 330 GBP expensive, it must be remembered that *Journal of Autoimmune Diseases *does not levy additional page or colour charges on top of this fee, which can easily exceed 330 GBP. With the article being online only, any number of colour figures and photographs can be included, at no extra cost.

## How will it work?

Authors will be asked to pay 330 GBP if their article is accepted for publication. Waiver requests will be considered on a case-by-case basis, by the Editor-in-Chief. Authors can circumvent the charge by getting their institution to become a 'member' of BioMed Central, whereby the annual membership fee covers the APC for all authors at that institution for that year. Current members include NHS England, the World Health Organization, the US National Institutes of Health, Harvard, Princeton and Yale universities, and all UK universities [11]. No charge will be made for articles that are rejected after peer review.

By providing a forum for Open Access, APC will enable *Journal of Autoimmune Diseases *to better serve the scientific community. We believe this change will benefit our readers and aid research in the field of autoimmunity.
